# Supplementary material for: How do social network models compare to all-to-all models for forecasting tuberculosis epidemics? A mathematical modeling study
Source: PLoS One. 2026 Apr 1;21(4):e0343421. doi: 10.1371/journal.pone.0343421 (PMC13042644; doi:10.1371/journal.pone.0343421)
Supplement: Attachment — Submitted filename: Review Report (PLOS ONE).docx [file pone.0343421.s001.docx]

- [The study presents the results of original research.](https://journals.plos.org/plosone/s/criteria-for-publication#loc-1)

The paper highlights social network attenuates the population-level impact of tuberculosis interventions and the study is demonstrated using mathematical model.

The work can be published in the journal PLOS ONE provided the following issues can be addressed.

1. System of ordinary differential equations is presented as partial differential equations. The notation of equations (1-5) need to be corrected.
2. The authors can check paper [29] on how system of ordinary differential equation is formulated and presented.
3. The schematic diagrams (A /B) are shows as figures under fig 2. The schematic diagrams must be shown before formulating the system of ordinary differential equations (ODE’s)
4. The figures are shown in different pages without caption. The captions are presented elsewhere. It is very difficult to follow the sequence of this paper.

- [Results reported have not been published elsewhere](https://journals.plos.org/plosone/s/criteria-for-publication#loc-2).

The results have not been published elsewhere, the authors need to put this ideas in a way which readability will be easy.

- [Experiments, statistics, and other analyses are performed to a high technical standard and are described in sufficient detail.](https://journals.plos.org/plosone/s/criteria-for-publication#loc-3)

The statistical results are presented very well and they were generated using R software.

- [Conclusions are presented in an appropriate fashion and are supported by the data](https://journals.plos.org/plosone/s/criteria-for-publication#loc-4).

The conclusion is presented in the correct scientific format and it validate the presented mathematical models.

- [The article is presented in an intelligible fashion and is written in standard English](https://journals.plos.org/plosone/s/criteria-for-publication#loc-5).

Yes, the article is written and presented in the correct English language.

- [The research meets all applicable standards for the ethics of experimentation and research integrity](https://journals.plos.org/plosone/s/criteria-for-publication#loc-6).

Yes

- [The article adheres to appropriate reporting guidelines and community standards for data availability](https://journals.plos.org/plosone/s/criteria-for-publication#loc-7).

I am comfortable with the report, it adheres to all the guidelines.

Final outcome: The paper can be published provided all the recommended corrections are adhered to.

Your Truly

Dr MC Kekana (12 June 2025)
