# Supplementary material for: How do social network models compare to all-to-all models for forecasting tuberculosis epidemics? A mathematical modeling study
Source: PLoS One. 2026 Apr 1;21(4):e0343421. doi: 10.1371/journal.pone.0343421 (PMC13042644; doi:10.1371/journal.pone.0343421)
Supplement: Attachment — Submitted filename: Response to Reviewers.docx [file pone.0343421.s005.docx]

**September 3, 2025**

**Re: Resubmission of Manuscript PONE-D-24-57786, Do social networks attenuate the population-level impact of tuberculosis interventions? A mathematical modeling study.**

Dear Dr. Mickael Essouma and Reviewers,

We would like to thank you for the constructive and insightful feedback on our manuscript. Below we provide a point-by-point response. Reviewer’s comments are reproduced in italics, and our responses follow in underlined regular text.

We hope that our revisions have addressed all comments and improved the manuscript. We thank the reviewers and editor once again for their thoughtful feedback.

Sincerely,

Masabho P. Milali, PhD

**Editor comments**

*1.Please ensure that your manuscript meets PLOS ONE's style requirements, including those for file naming. The PLOS ONE style templates can be found at*

[*https://journals.plos.org/plosone/s/file?id=wjVg/PLOSOne_formatting_sample_main_body.pdf*](https://journals.plos.org/plosone/s/file?id=wjVg/PLOSOne_formatting_sample_main_body.pdf) *and* [*https://journals.plos.org/plosone/s/file*](https://journals.plos.org/plosone/s/file)*? id=ba62/PLOSOne_formatting_sample_title_authors_affiliations.pdf*

Response: We now confirm that the resubmission was prepared using the official PLOS ONE style templates with corrected file naming conventions.

*2. Please note that funding information should not appear in any section or other areas of your manuscript. We will only publish funding information present in the Funding Statement section of the online submission form. Please remove any funding-related text from the manuscript.*

Response: We now remove all funding mentions from the main text and retain funding details only in the Funding Statement. (Lines 405 – 408)

*3. We note that the grant information you provided in the ‘Funding Information’ and ‘Financial Disclosure’ sections do not match. When you resubmit, please ensure that you provide the correct grant numbers for the awards you received for your study in the ‘Funding Information’ section.*

Response: We now reconcile Funding and Financial Disclosure details. (Lines 405-408)

4. Thank you for stating the following financial disclosure:

*“This work was supported by the Global Health Pilot Grant Program, Section for Global Health, Department of Population Health, NYU Langone Health.”Please state what role the funders took in the study. If the funders had no role, please state: "The funders had no role in study design, data collection and analysis, decision to publish, or preparation of the manuscript." If this statement is not correct you must amend it as needed. Please include this amended Role of Funder statement in your cover letter; we will change the online submission form on your behalf.*

Response: We now add: “The funders had no role in study design, data collection and analysis, decision to publish, or preparation of the manuscript”. (Lines 405-408).

*5. Please provide a complete Data Availability Statement in the submission form, ensuring you include all necessary access information or a reason for why you are unable to make your data freely accessible. If your research concerns only data provided within your submission, please write "All data are in the manuscript and/or supporting information files" as your Data Availability Statement.*

Response: We now state: “All data are in the manuscript”. (Lines 257-260, 418-421)

*Additional Editor Comments:*

*I comend the the authors for conducting a mathematical modeling study to assess whether the Barabasi-Albert scale-free mathematical model performs better than the all-in-model model when it comes to forecasting tuberculosis (TB) transmission patterns and hence epidemics in a population, given the high global burden of tuberculosis.*

Response: We thank the editor for the positive feedback

*However, there are important methodological and editing issues potentially limiting the reliability, readability and impact of the manuscript.*

*Major comments*

*1. Regarding the comment of reviewer 3 about established reporting guidelines not mentioned in the manuscript, consider complying with the EPIFORGE 2020 guidelines and report that you complied with those guidelines at the beginning of the Materials and Methods section. This entails the authors need to extend the Materials and Methods section in a way that each element required in the methods section according to the EPIFORGE 2020 guidelines would be provided in an individual sub-section. The sub-section "Ethical approval" of the Materials and Methods section needs to revised into "Ethical considerations" and moved to the end of the Materials and Methods section. The ethical considerations sub-section needs to include not only information about ethical approval, but also information about whether the authors complied with ethical rules for health research, although their research did not include human or animal participants. Plus, consider uploading an EPIFORGE 2020 checklist, based on Table 1 of*

*the article Pol;let etal. PLoS Med 18(10): e1003793. https://doi.org/10.1371/journal.pmed.1003793, as a supplemental material to showcase how you pragmatically complied with the EPIFORGE 2020 guidelines. See: Pollett S, Johansson MA, Reich NG, Brett-Major D, Del Valle SY, Venkatramanan S, et al. (2021) Recommended reporting items for epidemic forecasting and prediction research: The EPIFORGE 2020 guidelines. PLoS Med 18(10): e1003793.* [*https://doi.org/10.1371/journal.pmed.1003793*](https://doi.org/10.1371/journal.pmed.1003793)*.*

Response: We thank the editor for this important recommendation. The revised manuscript now complies with the EPIFORGE 2020 guidelines. At the beginning of the Materials and Methods section, we explicitly state our adherence to EPIFORGE, and the section has been restructured into individual sub-sections following the guideline items. The former “Ethical approval” subsection has been revised to “Ethical considerations” and moved to the end of the Materials and Methods. This subsection now specifies compliance with ethical standards for modeling research, even though no human or animal participants were involved. An EPIFORGE 2020 checklist, based on Table 1 of Pollett et al. (2021), has also been prepared and uploaded as supplemental material to document our compliance. Changes reflected in lines 92 - 260

*2. Manuscript's title. Going through the introduction, I wonder whether the title "Are social networks better than all-in-all models for forecasting tuberculosis epidemics? A mathematical modelling study" does not resonate more with the work you conducted and reported in this manuscript.*

Response: While we agree with your proposed framing, we revised the title to remove the phrase “better than” to avoid implying superiority between models, since the study was not fit to empirical data and therefore cannot establish whether one model is “better.” Instead, our comparison highlights how different assumptions about contact structure influence projections. The revised title is:

**“How do social network models compare to all-to-all models for forecasting tuberculosis epidemics? A mathematical modeling study.”**

We believe this phrasing captures the comparative nature you recommended while avoiding unintended claims of superiority, and ensures the title remains scientifically accurate, testable, and aligned with the scope of our study (Lines 1–2)

*3. Abstract. Consider adjusting its content with that of the revised main manuscript.*

Response: Abstract rewritten to match updated sections of the manuscript. (Lines 12-26)

*4. Consider inserting keywords just after the abstract.*

Response: Keywords inserted just after the abstract. (Lines 29-30)

*5. Introduction. I suggest revising the introduction to better inform readers why you conducted this study and provide the study purpose in a clearer way. Accordingly, you need to extend the first paragraph with more details about the definition of tuberculosis (using updated terms: see Garcia-Basteiro et al. Lancet Glob Health. 2024 May ; 12(5): e737–e739. doi:10.1016/S2214- 109X(24)00083-4.), tuberculosis subtypes (based on organ involvement, drug sensitivity and disease activity), the burden of drug-sensitive and drug-resistant tuberculosis, including details about the impact of tuberculosis treatment and vaccination on the control of the TB epidemic across low and high endemicity TB settings around the world (Chihota et al. Drugs (2025) 85:127–147 https://doi.org/10.1007/s40265-024-02131-3 and DOI 10.3389/fpubh.2024.1408316). You would then go on to describe patterns of tuberculosis transmission across low and high endemicity tuberculosis areas around the world, including risk factors for transmission and how mathematical models have helped decipher the patterns of*

*tuberculosis transmission and hence TB epidemic around the world so far. (Chihota et al. Drugs (2025) 85:127–147 https://doi.org/10.1007/s40265-024-02131-3, DOI 10.3389/fpubh.2024.1408316 and Lo et al. Epidemics 39 (2022) 100570) While doping this, you would show how all-in-all models have mainly contributed to this endeavour whereas there is a considerable knowledge gap about how and why the Babarasi-Albert scale-free social network model could better decipher the patterns of TB transmission (in which population specifically?) and hence better forecast and prevent TB epidemics in (some or all) populations. You need to do this with more details than actually but in a more simplistic way. See how Wikipedia has commented on the Barabasi-Albert scale-free social network model: https://en.wikipedia.org/wiki/Barab%C3%A1si%E2%80%93Albert_model)Following this, the authors would usher in the study purpose.*

Response: The Introduction has been expanded to incorporate the requested elements, including updated definitions of tuberculosis (Garcia-Basteiro et al., 2024), tuberculosis subtypes, and the burden of drug-sensitive and drug-resistant TB. We have also added details on the impact of treatment and vaccination across high- and low-burden settings, as well as on patterns of TB transmission and the contribution of mathematical models to understanding epidemic dynamics (Chihota et al., 2025; Lo et al., 2022). The knowledge gap regarding the Barabási–Albert scale-free model is now highlighted, and the study’s purpose has been clarified. (Lines 32–64)

*6. Materials and Methods. Along with my comment above raising the need to subdivide the Methods section into many sub-sections to improve its readability, I have other comments about this section. What TB subtypes did you incorporate in the models: all-type tuberculosis as it seems in the discussion section? Consider specifying in this section. Why did you assume that "the same underlying progression" and treatment and vaccine efficacy would be observed in both studied models? Would the TB epidemic progress in the same way in both scenarios in real life? What did you mean buy "steady-state" in the context of this study? Consider specifying. It is in the discussion section that you first specified that the population incorporated in the models was not representative of any specific setting or geographic location , but it is in this section that you need to clearly specify that and that you did not stratify the population into low and highest-risk (e.g., under-five children, older adults, immunocompromised (especially HIV/AIDS) patients, incarcerated individuals, migrants [especially refugees, asylum seekers, war/torture/crisis survivors]) and most visible community members. Along this line, the authors need to more precisely enumerate these population groups when addressing the study limitations because the TB epidemic is virtually different in these population groups than in other lower-risk population groups. Furthermore, when calling expanded modelling efforts incorporating real-world social network complexities, the authors would like to more precisely address the potential influence of most visible community members in TB transmission around the world (https://en.wikipedia.org/wiki/Barab%C3%A1si%E2%80%93Albert_model), given that in the current context of globalization, most people encounter Mycobacterium tuberculosis at some point of their life and keep it in their bodies throughout their lifetime. See Chihota et al. Drugs (2025) 85:127–147 and current reference 29. The age group of the represented population was not mentioned in this section. There is a need for a sub-section on definitions of terms (e.g., incidence, prevalence, treatment recovery/efficacy, treatment adherence, imperfect adherence, treatment efficacy, vaccine efficacy and vaccine coverage) in this section given the strong comments you made in the discussion section using some of those terms. What treatments did you consider in the models? Same question for vaccines knowing that BCG remains the only certified TB vaccine to the best of my knowledge. See https://doi.org/10.1371/journal.pone.0317233. It should also be clear that the BCG is mainly recommended in young populations owing to its limited efficacy in adults, and BCG mass vaccination is uncommon in low endemicity areas whereas the authors claim that the model did not consider any geographic boundary. Can you ponder about this in this section and in the limitations statement of the discussion section? What guided the choice of treatment recovery rate (20, 30, 40, 50 percent), vaccine coverage rate (40, 50, 60, 70) and time schedule thresholds (400 days to reach the equilibrium, 300 days after the start of the treatment (or 700 days after launching the model) to estimate the impact of interventions? What did you mean by "model reaching an equilibrium"? Why did you need the equilibrium to be reached to simulate the impact of TB interventions? Could you elaborate more on the procesd of simulating the impact of interventions? All these precision's are warranted. You performed sensitivity analyses, but you also need to provide information on results calibration and validation, at least in the limitations statement of the discussion section. See Lo et al. Epidemics 39 (2022) 100570).*

Response: We carefully revised the Materials and Methods to address each point raised.

- *Along with my comment above raising the need to subdivide the Methods section into many sub-sections to improve its readability*

Response: We now explicitly state compliance with the EPIFORGE 2020 guidelines at the start of the Methods (line 93). The section has been restructured into EPIFORGE-compliant subsections: Study Overview, Model Structure and Assumptions, Definitions of Terms, Parameterization and Calibration, Intervention Scenarios, Outcome Measures, Analysis Methods, Ethical Considerations, and Reproducibility (line 92–260).

- *What TB subtypes did you incorporate in the models: all-type tuberculosis as it seems in the discussion section?*

Response: We now clarify that the models represent all-form TB without stratification by organ involvement or drug resistance. This is stated explicitly in the Study Overview (line 109–111)

- *Why did you assume that "the same underlying progression" and treatment and vaccine efficacy would be observed in both studied models? Would the TB epidemic progress in the same way in both scenarios in real life?*

Response: We now explain that assuming identical disease progression, treatment efficacy, and vaccine efficacy across both models is a deliberate simplification to isolate the effect of contact structure. We note that in real-world scenarios, TB epidemics may not progress identically under homogeneous versus networked contact structures, but this difference is precisely the focus of our study. This point is reflected in the revised Methods (lines 201–205) and reinforced in the Discussion (lines 374–379).

- *What did you mean by "steady-state" in the context of this study?*

Response: We now define steady-state as “a stable epidemiological condition in which TB incidence and prevalence remain constant over time in the absence of new interventions” (Definitions of Terms, lines 184–186).

- *It is in the discussion section that you first specified that the population incorporated in the models was not representative of any specific setting or geographic location , but it is in this section that you need to clearly specify that and that you did not stratify the population into low and highest-risk (e.g., under-five children, older adults, immunocompromised (especially HIV/AIDS) patients, incarcerated individuals, migrants [especially refugees, asylum seekers, war/torture/crisis survivors]) and most visible community members. Along this line, the authors need to more precisely enumerate these population groups when addressing the study limitations because the TB epidemic is virtually different in these population groups than in other lower-risk population groups. Furthermore, when calling expanded modelling efforts incorporating real-world social network complexities, the authors would like to more precisely address the potential influence of most visible community members in TB transmission around the world (https://en.wikipedia.org/wiki/Barab%C3%A1si%E2%80%93Albert_model), given that in the current context of globalization, most people encounter Mycobacterium tuberculosis at some point of their life and keep it in their bodies throughout their lifetime. See Chihota et al. Drugs (2025) 85:127–147 and current reference 29. The age group of the represented population was not mentioned in this section.*

Response: We now clarify in the Methods that the simulated population was not stratified by age, risk, or sociodemographic factors, and instead represented a generic mixed-age adult cohort (Lines 150–154). In the Discussion (Limitations – lines 368 - 373), we expand on this point to note that the model does not capture heterogeneity across high-risk groups such as children under five, older adults, immunocompromised individuals (including those with HIV/AIDS), incarcerated populations, or migrants. We also highlight that TB epidemics can differ substantially across these groups and that real-world transmission may be disproportionately influenced by highly connected “visible” community members.

- *There is a need for a sub-section on definitions of terms (e.g., incidence, prevalence, treatment recovery/efficacy, treatment adherence, imperfect adherence, treatment efficacy, vaccine efficacy and vaccine coverage) in this section given the strong comments you made in the discussion section using some of those terms.*

Response: In line with this suggestion, we have added a dedicated Definitions of Terms subsection in the Methods (lines 175–187). This subsection provides clear definitions of key terms used throughout the manuscript, including incidence, prevalence, treatment recovery rate, treatment efficacy, treatment adherence, imperfect adherence, vaccine efficacy, vaccine coverage, and steady-state.

- *What treatments did you consider in the models? Same question for vaccines knowing that BCG remains the only certified TB vaccine to the best of my knowledge. See https://doi.org/10.1371/journal.pone.0317233. It should also be clear that the BCG is mainly recommended in young populations owing to its limited efficacy in adults, and BCG mass vaccination is uncommon in low endemicity areas whereas the authors claim that the model did not consider any geographic boundary. Can you ponder about this in this section and in the limitations statement of the discussion section?*

Response: We now clarify in the Methods that treatment is modeled as a generic composite regimen with efficacy assumptions aligned with WHO-reported outcomes for drug-sensitive TB, rather than specific drug classes (lines 232–237). Vaccination is modeled using a generic sterilizing vaccine profile to enable scenario exploration and does not explicitly represent BCG (lines 226–232). We further note that BCG remains the only licensed TB vaccine, that its efficacy is limited in adults, and that BCG mass vaccination is uncommon in low-endemicity settings. These clarifications are included in both the Methods and the Limitations sections to emphasize that the modeling approach is not geography-specific and should be interpreted as a structural comparison rather than a setting-specific forecast (lines 350 – 353; 379 – 385).

- *What guided the choice of treatment recovery rate (20, 30, 40, 50 percent), vaccine coverage rate (40, 50, 60, 70) and time schedule thresholds (400 days to reach the equilibrium, 300 days after the start of the treatment (or 700 days after launching the model) to estimate the impact of interventions?*

Response: We now clarify in the Methods that the treatment recovery rate increases (20%, 30%, 40% or 50%) and vaccine coverage levels (30%, 40% 50%, and 70%) were selected to span plausible programmatic and clinical ranges reported in previous TB modeling and empirical studies (references 29, 30) (lines 226–238). The time schedule was designed to allow a 400-day burn-in period for the models to reach equilibrium, followed by a 300-day post-intervention period (through day 700 of the total simulation). This ensures that intervention impacts are evaluated after stabilization rather than during initial transients, and captures medium-term effects while minimizing excessive overlap with longer-term stochastic drift (lines 238–242).

- *What did you mean by "model reaching an equilibrium"? Why did you need the equilibrium to be reached to simulate the impact of TB interventions?*

Response: We now define *steady-state* (equilibrium) in the Definitions of Terms subsection, clarifying it as ‘a stable epidemiological condition in which TB incidence and prevalence remain constant over time in the absence of new interventions’ (lines 183–187). We require the models to reach this equilibrium before simulating interventions to ensure that intervention effects are measured relative to a stable baseline rather than during initial transient dynamics. This clarification is reflected in the revised Methods, (lines 220-227; 238–243)

- *Could you elaborate more on the process of simulating the impact of interventions?*

Response: We now expand the Intervention Scenarios subsection to clarify the process of simulating interventions. After a 400-day burn-in period to allow the models to reach equilibrium, interventions were introduced as either vaccination or treatment scale-up. Vaccination was implemented as a one-time mass campaign in which a specified fraction of the susceptible population was transferred to the vaccinated compartment (lines 119–120; 134-139; 220-243). Treatment interventions were modeled as increases in the recovery rate from active TB by 20%, 30%, 40% or 50% over baseline (lines 228–237). Intervention impacts were quantified as the percent reduction in the number (prevalence) of infectious individuals at 300 days post-intervention (day 700 of the simulation) relative to a no-intervention baseline; time-series trajectories were also compared to show dynamics (lines 232–247)

*7. Results. Could you separate the presentation of results of the all-in-all model from that of the Barabasi-Albert scale-free social network model? Consider further adjudicating the results in agreement with revisions of the Materials and Methods section.*

- Response: Results are now restructured into model-specific subsections (All-to-All Model Results and BA Scale-Free Network Model Results) and adjusted to align with revisions made in the Materials and Methods section (lines 261 – 327)

*8. Discussion and conclusion sections. I appreciate the enumeration of study limitations.*

*However, the authors should consider specifying the impact of each major limitation on the interpretation of satudy results. Fpor example, when stating that "our study did not focus on specific settings or geographical regions.", they would better complete the statement by stating something like:@ "because patterns of TB epidemic vary across low and and high endemicity TB settings." Also, besides the limitations highlighted in the current manuscript, the authors need to specify whether this study only considered drug-sensitive tuberculosis as in previous with tuberculosis modelling studies, in a context where the relative contribution of drugsensitive and drug-resistant tuberculosis to the overall burden of tuberculosis in real-life may not be well known (Song HW, Tian JH, Song HP, Guo SJ, Lin YH and Pan JS (2024) Tracking multidrug resistant tuberculosis: a 30-year analysis of global, regional, and national trends. Front. Public Health 12:1408316. doi: 10.3389/fpubh.2024.1408316). The non incorporation of social determinants (notably in the social network) was also a major limitation given that their*

*influence on population incidence of tuberculosis may be more important than that of TB*

*treatment. See lange et al. Pathog Immun. 2025 Mar 2;10(2):1-45. doi: 10.20411/pai.v10i2.791. At the end of this manuscript, the population group in which this study would best help prevent the spread of the Tb epidemic is unclear. Likewise, the TB subtype (at least based on organ involvement pulmonary and extrapulmonary TB) this study would best help prevent is unclear. Even if the authors considered all-form tuberculosis, the risk for incident extrapulmonary TB is more common in under-five children and immunocompromised persons than in other population groups whereas pulmonary tuberculosis (at least pulmonary tuberculosis infection [Garcia-Basteiro eyt al. https://doi.org/10.1016/s2214-109x(24)00083-4]) might be more evenly distributed among immunocompetent and immunocompromised persons. See https://doi.org/10.3390/jor1020015 and Guzmán-Beltrán, S.; Bobadilla, K.; Santos-Mendoza, T.; Flores-Valdez, M.A.; Gutiérrez-González, L.H.; González, Y. Human Pulmonary Tuberculosis: Understanding the Immune Response in the Bronchoalveolar System. Biomolecules 2022, 12, 1148. https://doi.org/10.3390/biom12081148. These issues need to be addressed in the discussion and conclusion sections.*

- Response: We have revised the Discussion and Conclusion sections to address each of these points. The Limitations section now specifies the interpretive impact of each major limitation (347 - 391). We have further clarified that the model did not stratify by age or risk group, and therefore the results are most applicable to pulmonary TB in mixed adult populations. Extrapulmonary TB, more common among children and immunocompromised individuals, was not explicitly modeled (lines 368-373). Finally, the Conclusion is now revised to reflect these clarifications, emphasizing that the findings apply primarily to pulmonary TB in generic adult populations and should not be generalized to setting- or subgroup-specific forecasts without further calibration (lines 398-405)

*Minor comments*

*1. Consider formatting the manuscript as recommended in PLOS One author guidelines,*

*including the references.*

- Response: The manuscript has been reformatted in accordance with PLOS ONE author guidelines, including the reference style.

*2. Some language editing is warranted. For instance, I advise against using the words "predict" and "forecast" in the results section, but rather providing results of model simulation. Then, in the discussion section, you would make statements like: "Results of the all-in-all model would allow the prediction or forecasting of ....in a scenario where an infected individual may transmit TB to any individual of the same population, whereas results of the Barabasi-Albert scale-free social network would allow the prediction of .... in a scenario where an infected individual would transmit TB only to their social contacts.".*

- Response: The Results section was revised to consistently use “estimates” and “simulations” instead of “predict” or “forecast.” Forecasting language was moved appropriately to the Discussion, as suggested

*3. References need to be updated and strengthened.*

- Response: References are now updated with recent key citations, including Garcia-Basteiro et al. (2024), Chihota et al. (2025), Lo et al. (2022), Song et al. (2024), and Lange et al. (2025), to ensure that the Introduction, Methods, and Discussion are supported by the most current evidence.

*4. Consider numbering the manuscript's lines and sending figures and tables at the end of the manuscript to ease the assessment of the next version of the manuscript.*

- Response: Line numbering has been added, and all figures and tables have been placed at the end of the manuscript in accordance with PLOS ONE formatting requirements.

*5. Where are statements about conflicts of interests and data availability that should be found at the end of the manuscript before the reference section?*

- Response: We have added at the end of the manuscript before the References section, following journal requirements. The Data Availability statement specifies that all model equations and parameter values are provided in the manuscript and that code is available at the GitHub repository (lines 423-426). The Conflict of Interest statement confirms that the authors have no competing interests. (lines 420-421)

6*. Where in the manuscript did you state that there is a preprint for this manuscript*

*(*[*https://doi.org/10.21203/rs.3.rs-3283210/v1*](https://doi.org/10.21203/rs.3.rs-3283210/v1)*)?*

- Response: The preprint DOI (https://doi.org/10.21203/rs.3.rs-3283210/v1) has been added at the end of a manuscript to ensure transparency (lines 427 – 430)

**Reviewer #1**

*The work can be published in the journal PLOS ONE provided the following issues can be addressed:*

1. *System of ordinary differential equations is presented as partial differential equations. The notation of equations (1-5) need to be corrected.*

Response: The equations have been revised to use proper ordinary differential equation (ODE) notation, correcting the earlier presentation that appeared as partial differential equations (lines 163–175).

2. *The authors can check paper [29] on how system of ordinary differential equation is formulated and presented.*

Response: We have consulted the recommended paper (Liu et al., 2014) and aligned our equation notation and presentation with standard ODE formulations, as suggested (lines 163–175).

3. *The schematic diagrams (A /B) are shows as figures under fig 2. The schematic diagrams must be shown before formulating the system of ordinary differential equations (ODE’s)*

Response: The schematic diagrams (panels A and B) have been repositioned to appear before the system of equations, so that the model structure is clearly described visually before the mathematical formulation (lines 140 – 176)

4. *The figures are shown in different pages without caption. The captions are presented elsewhere. It is very difficult to follow the sequence of this paper.*

Response: Each figure is now presented on a single page with its caption directly below

**Reviewer #2**

*I recommend that the authors revise their manuscript while addressing each of*

*the following points:*

- *The model is not tailored to specific geographic or demographic settings. Can the authors relate their work to real world epidemiological data?*

Response: We now clarify in the Methods that the models are not tailored to specific geographic or demographic settings but instead represent a generic mixed-age adult cohort. In the Discussion (Limitations), we explicitly note that this limits transferability because TB epidemic patterns differ across low- and high-burden settings, and that future work should calibrate to empirical data such as WHO surveillance or PHIA surveys for local applicability (lines 149–154; 346–357).

- *The authors should include in the discussions section a few lines about the possibility of exploring additional network topologies (e.g., small-world or community-based) to explain how structural differences influence intervention outcomes.*

Response: We have acknowledged in the Limitations that using a single Barabási–Albert scale-free model may not fully capture the diversity of real-world social interactions. We have added that other network structures, such as small-world or community-based topologies, could provide additional insight into how social structure influences intervention outcomes (lines 357 - 364).

- *Instantaneous vaccination is an issue of concern in the model. Real life scenarios would have gradual roll-out of vaccination. I suggest to address this issue as well in the discussions.*

Response: We have revised the Discussion to note that our assumption of a one-time mass vaccination campaign is a simplification, and that real-world vaccination rollouts occur gradually. We have emphasized that future modeling should incorporate phased or delayed rollouts to better approximate real-world implementation (lines 378–385).

- *The issue of vaccine availability as well as timing of introduction is discussed and modelled in the literature. One such study that the authors can refer to on their introduction or in their discussion while addressing the previous points is the following:https://doi.org/10.1371/journal.pone.0267840*

Response: We have added the suggested study by Deeb et al. (2022) in the Introduction and noted how vaccine availability and timing influence intervention outcomes. These changes are reflected in the Introduction (lines 52–57)

- *Assumption of 100% vaccine efficacy is very ideal and usually not true. I suggest that in the model results, the authors consider a bracket of efficacies, for example 80-85-90-95% scenarios, with corresponding plots and comparisons. This could be done 1 figure, then move to a particular benchmark in the rest of the results, maybe 90% per se.*

Response: We now clarify in the Methods that assuming 100% vaccine efficacy was intentional to isolate the effect of contact structure without introducing confounding from waning immunity or breakthrough infections (lines 133–140). In the Discussion, we have acknowledged that real-world vaccines are likely to have partial efficacy, and have noted that bracketed efficacy scenarios (e.g., 80–95%) are important for future work once candidate TB vaccines are available (lines 378–385).

- *Social determinants affecting treatment uptake and exposure risk were not considered. Explain how such factors could improve projections and guide equity-oriented interventions. I recommend a major revision to specifically address these points, and, if treated well, the paper would be suitable for consideration afterwards.*

Response: We have expanded the Limitations to acknowledge that social determinants such as poverty, stigma, and healthcare access strongly influence TB transmission and treatment uptake, potentially outweighing biomedical interventions. We have cited Lange et al. (2025) to emphasize this point and noted that excluding these factors limits interpretation to biomedical interventions (lines 364–367).

**Reviewer # 3**

*I have identified several issues that need to be addressed to meet PLOS ONE publication*

*standards:*

*Major issues:*

1. *The manuscript lacks mandatory elements for PLOS ONE publication. For example, there is no data availability statement indicating where model code, parameter values, and analysis scripts will be made available. There is also no reference to established reporting guidelines for mathematical modeling studies. The ethics statement, while appropriate for a modeling study, should be expanded to specifically mention data sources used and confirm no human subjects were involved.*

Response: We have added a Data Availability statement confirming that all model equations and parameter values are in the manuscript and that the R code is publicly available via GitHub (lines 256–261; 422-427). We also have structured the Methods in compliance with the EPIFORGE 2020 reporting guidelines and explicitly noted compliance at the start of the section (line 93). The Ethical Considerations subsection has been expanded to confirm that no human subjects or animals were involved, and that only simulated data were used (lines 253–256).

2. *It appears the author might have made some claims that extend well beyond what the modeling results can support, in the discussion section. The claims include:*
a) *Statement that findings "call into question the sufficiency of resources currently allocated to global TB programs"*
b) *Assertion that "even greater resources may be necessary"*
c) *Call for "Redoubled efforts are needed to invest in more effective and accessible vaccination..." These statements read more like policy advocacy than scientific conclusions. The manuscript would be strengthened if the authors instead focused on the specific modeling implications with quantified results. For example, instead of making broad resource allocation claims, the authors could indicate that their findings suggest social network models reduce projected efficacy of sub-optimal interventions by X–Y percentage points, potentially affecting the feasibility of current targets when interventions have limited coverage*

Response: The Discussion has been revised to focus on modeling implications rather than policy advocacy. Phrases such as “call into question the sufficiency of resources”, “even greater resources may be necessary”, and “redoubled efforts” were removed or replaced with precise statements about the magnitude of differences between models. The revised text emphasizes interpretation of simulation results rather than prescriptive policy (lines 329-392).

3. *Although important limitations were acknowledged, the authors did not explain how the limitations affected the reliability of their study conclusions (including policy recommendations) or connect the limitations to the validity of their study findings.*

Response: The Limitations section has been expanded to specify the impact of each limitation on interpretation. Changes are reflected in lines 346 - 392.

***Minor issues:***

4. *There are language and clarity problems throughout the manuscript:*
a) *Several sentences are unnecessarily complex and should be simplified (an example is the second sentence in the abstract (“We compared the impact of TB treatment and vaccination in an all-to-all compartmental model versus a social network model that…). This sentence tries to pack too much information into one complex statement with multiple clauses, making it harder to follow*

Response: The second sentence in the abstract, along with similar long sentences, has been simplified by breaking it into shorter sentences for easier readability.

b) *Inconsistent formatting of "all-to-all" (sometimes in quotes, sometimes not)*

Response: Formatting of all-to-all is now standardized throughout the manuscript (without quotation marks).

c) *Ambiguous phrasing like "plateau relative to the 'all-to-all' model". The authors should clarify whether the incidence plateaued at higher levels in the network model.*

Response: Phrasing such as “plateau relative to the ‘all-to-all’ model” is clarified to state explicitly that incidence plateaued at higher levels in the BA network model (lines 281–286; 302-309).

d) *Informal language like "Redoubled efforts" should be replaced with more precise academic language*

Response: Informal wording such as “redoubled efforts” has been replaced with more precise academic phrasing.

5. *The discussion should begin with a summary of what the research achieved relative to the authors’ stated aims, rather than immediately diving into broad implications.*

Response: The Discussion now begins with a concise summary of the study’s objectives and main findings before moving to broader implications (lines 328–339).

6. *The conclusion lacks precision in the use of implied statements. For example the phrase "large implications" needs quantification. It would be better if he authors could specify the actual magnitude of divergence they observed. This would give readers concrete understanding of when these modeling differences matter most.*

Response: The Conclusion has been revised to replace vague phrases such as “large implications” with quantified statements (lines 392–404).

**Reviewer #4**

*Methods: Model development*

*I would suggest the authors review the model equations and diagram:*

1. *The model diagram and equations specify that those persons that leave each compartment due to death (either natural or disease related) re-enter the susceptible compartment. This cannot be, since persons that leave compartments due to death leave the system entirely, despite the birth and death rates being equal.*

Response: The model structure and equations have been revised so that all deaths (natural or TB-related) exit the system into a death sink, and do not re-enter the susceptible compartment. Births replenish the susceptible pool separately to maintain constant population size (lines 114–175).

2. *The inflow into the S compartment and outflow from S to V in diagram B is not reasonable. Persons are recruited or born into the S compartment at a rate (the birth/recruitment rate). From this compartment a proportion can be vaccinated (lambda) at the vaccination rate and enter the V compartment. The first aspect I suggest the authors revisit is the inflow into the S compartment. The (1-lambda) does not fit since you are saying that those of the S compartment that were not vaccinated are recruited into S. All persons enter S unvaccinated. Thereafter they either proceed to V or the remaining unvaccinated proceed along to E or die out of the S compartment. The second aspect is the movement from S to V. The vaccination rate is indicated to be the death/birth rate. Is this a valid assumption? The third aspect is the movement of the remaining S population to E and further. Not all persons proceed along that route, but only those that were not vaccinated, i.e., the proportion (1-lambda). A further question is whether the authors assume the vaccinated individuals incur life-long immunity or whether they loose protection at some point and re-enter the S compartment?*

Response: The diagram and equations were updated to clarify that all individuals enter the susceptible compartment unvaccinated, and from there a fraction λ is vaccinated and transferred to V at the time of intervention. We also clarified that this was modeled as a one-time campaign rather than a continuous birth-vaccination flow. The assumption of lifelong immunity in V was explicitly stated, reflecting a hypothetical sterilizing vaccine scenario (lines 114–175; Fig. 2)

3. *An important assumption not provided within the model description is that it is assumed that disease related death only occurs among those with active TB, i.e., in the I compartment. Further, despite disease related death from I, do you further assume that those with active TB do not undergo natural death?*

Response: We have clarified that TB-related mortality was applied only to individuals in the infectious compartment (I), and that these individuals also experienced natural mortality. All other compartments experienced only natural mortality (lines 128–130).

4. *The model equations are missing some terms, namely in equation 1 and 2, the vaccination rate is not included in the equation. Equation 3, (1-p)*sigma*E is missing.*

Response: We thank the reviewer for this careful reading. In the revised version, we ensured vaccination terms are explicitly included in equations (1) and (2). Regarding equation (3), the (1−p)σE term was not omitted but was previously simplified into the transition structure during drafting, which may have created confusion. In the revised manuscript, the equations are now written in full form to explicitly display all transition terms, including (1−p)σE, for clarity (lines 164 – 175).

5. *It would be of benefit for the readers -especially those without an infectious disease modelling background - that you provide some explanation of a scale-free network model - particularly what the major assumptions are- and also clearly outline your process of calibration. Further, although the link to the code repository is provided, it would be beneficial for the readers to get a brief overview of what the code actually does, generating the figures provided.*

Response: We now provide a plain-language explanation of the Barabási–Albert (BA) scale-free model in the Study Overview and Objectives subsection (lines 94–107), highlighting its major assumption of preferential attachment and the resulting hubs with many more connections than most nodes. To further aid readers without a modeling background, we included Fig. 1 (lines 99 -107) to visually illustrate differences between scale-free and all-to-all contact structures. In addition, we expanded the Parameterization and Calibration subsection to describe how β_net was calibrated against β_all to ensure comparable epidemic trajectories (lines 187–201).

6. *Model equations are formulated as PDEs pointing towards equations that are functions of more than one variable. Time is clearly specified, but what is the other? The authors further specify the use of 'deSolve' which primarily works with ODEs without some creative extension. Should the latter be the case, please include a clear description of the methodology behind the extension.*

Response: The equations have been reformatted explicitly as ordinary differential equations (ODEs), clarifying that time (t) was the only independent variable. We note that ‘deSolve’ was used for ODE integration, not PDEs (lines 164-175; 248-253).

7. *The methods would benefit from a clear description of the model (as outlined in the model diagram) including the assumptions defining the transitions and parameter selections.*

Response: The Model Structure and Assumptions subsection has been expanded to describe transitions, parameter selections, and the constant-population assumption in detail, in line with the revised diagram and equations (Methods – Model Structure and Assumptions, lines 114–175; Table 1).

*Minor errors:*

1. *Under Model development, line 3: "emplying" should be "employing"*

Response: The typographical error “emplying” has been corrected to “employed” (Methods – Analysis Methods, line 250).

**Reviewer # 5**

*The author need to provide point-by-point response or rebuttal to my observations listed below.*

- *Include some major outcomes in the abstract.*

Response: We have revised the abstract to summarize the key quantitative outcomes, highlighting the differences between the all-to-all and BA scale-free models (lines 13 – 27).

- *To extend this idea to the utilization of nanotechnology in the assumed model consult following works:* [*http://dx.doi.org/10.1016/j.rinp.2024.107863*](http://dx.doi.org/10.1016/j.rinp.2024.107863)*,* [*http://dx.doi.org/10.1016/j.ijheatfluidflow.2024.109322*](http://dx.doi.org/10.1016/j.ijheatfluidflow.2024.109322)*, http://dx.doi.org/10.1038/s41598-023-48364-2,* [*https://doi.org/10.1016/j.csite.2024.104178*](https://doi.org/10.1016/j.csite.2024.104178)*, http://dx.doi.org/10.1177/14613484231216198,* [*http://dx.doi.org/10.1016/j.asej.2023.102316*](http://dx.doi.org/10.1016/j.asej.2023.102316)

Response: We appreciate this suggestion. Our study is intentionally designed to remain as simple as possible in order to isolate the impact of contact structure on intervention effects. As such, we did not model specific products, drug formulations, or delivery mechanisms, whether for drug-sensitive or drug-resistant TB, or nanotechnology-based interventions. We agree that nanotechnology offers promising opportunities for improving TB treatment, diagnosis, and vaccination, and view this as a valuable avenue for future research building upon our work.

- *Does the manuscript address an important gap in tuberculosis modeling by comparing "all-toall"and social network-based transmission structures?*

Response: To the best of our knowledge, no previous study has directly compared an all-to-all model with a social network (Barabási–Albert) model while holding all other assumptions constant. Our study isolates the role of contact structure itself by ensuring that disease progression, treatment, and vaccine parameters were identical across frameworks. This design highlights how the homogeneous mixing assumption in all-to-all models can overestimate the population-level impact of interventions when compared with the heterogeneous contact structure of network-based models (lines 75 – 91).

- *How novel is the direct comparison between these two modeling frameworks, particularly when using identical TB assumptions?*

Response: We have clarified in the Introduction that this study addresses an important gap in TB modeling by directly comparing intervention impacts across all-to-all and Barabási–Albert social network frameworks under harmonized assumptions. To our knowledge, no prior study has systematically isolated the role of contact structure by holding natural history, treatment, and vaccine parameters constant between frameworks. This novelty highlights how differences in contact assumptions (homogeneous vs. heterogeneous) alone can alter projected intervention impacts (lines 75–91).

- *Could the authors better explain how this work differs from previous modeling studies of TB or other infectious diseases?*

Response: We have clarified in the Introduction that prior TB modeling has largely used compartmental all-to-all frameworks, while this study directly compares those with a scale-free network under identical assumptions to reveal when model structure matters most (lines 75–91).

- *The study assumes 100% vaccine efficacy in many simulations. Can the authors justify this assumption in the context of current vaccine development?*

Response: In the Methods, we have explained that vaccine efficacy was assumed to be 100% as a deliberate simplification to isolate the influence of contact structure without added complexity from waning immunity or breakthrough infections (Methods – Model Structure, line 134–139). In the Discussion, we have acknowledged that this does not reflect real-world TB vaccine candidates and described partial efficacy scenarios as a priority for future work (Discussion – Limitations, line 378–385).

- *Have the authors considered including scenarios with more realistic vaccine efficacies (e.g.,50–70%) or “take” rates?*

Response: We now clarify that we did not include bracketed efficacy scenarios in this study because our primary objective was to isolate the effect of contact structure. Modeling <100% efficacy would have introduced additional variables, such as waning immunity, partial protection, or heterogeneous “take” rates, that could confound interpretation of how network versus homogeneous mixing assumptions alone influence intervention impact. By fixing efficacy at 100%, we ensured that any differences observed between models were attributable solely to contact structure. We now explicitly acknowledge in the Limitations that exploring partial efficacy scenarios is critical for future work, particularly once candidate vaccine trial data become available (Discussion – Limitations, lines 378–385; 401-404).

- *How were the transmission parameters (β) calibrated to ensure that both models produce similar baseline TB prevalence?*

Response: We have clarified in Methods that β_all was fixed at a published per-capita value, while β_net was calibrated to reproduce the same baseline prevalence and incidence as the all-to-all model. This ensured comparability across frameworks despite different contact structures (Methods – Parameterization and Calibration, line 188–210).

- *What justifies the specific prevalence level used for calibration?*

Response: Calibration was guided by aligning both models with typical prevalence and incidence values reported in high-burden settings, as recommended in prior TB modeling studies [Methods – Parameterization and Calibration, line 188–220).

- *Would sensitivity analysis around baseline prevalence assumptions add robustness to the findings?*

Response: While this study varied network degree and random seeds in sensitivity analysis, we note in the Limitations that varying baseline prevalence assumptions is an important next step to test the robustness of relative differences across models (Discussion – Limitations, line 366–392).

- *The conclusions follow from the presented results. I suggest that it is betters is authors can provide the future research prospective at the end of article.*

Response: The Conclusion was expanded to include future priorities, such as incorporating drug-resistant TB, social determinants, alternative network structures, and vulnerable population stratification (Conclusion, line 386–392).

**Reviewer #6**

*In particular, there are concerns related to key model assumptions and their biological plausibility. My specific concerns and suggestions are outlined below:*

1. *A more detailed explanation of the model is necessary to make it accessible to readers from various disciplines. The numerical simulation is presented over a span of more than 700 days, which cannot be considered a particularly short time frame. Is it essential to assume that the total population remains constant over this long time span? The current model appears unnecessarily complex primarily to preserve this assumption. There are much simpler and more straightforward ways to maintain a constant population without introducing such complications. It seems challenging—perhaps even impossible—to provide a detailed and biologically sound explanation of the current version of the transfer diagram and, by extension, the model itself. For example, the biological meaning of the last term in model equation (1) is unclear in the context of TB transmission.*

Response: We have expanded the Model Structure and Assumptions subsection to explain the constant-population assumption. Births replenish the susceptible compartment at the same rate as deaths across all compartments, ensuring population size remains fixed. This is chosen as a simplification to isolate the impact of contact structure, while acknowledging in the Discussion that demographic change could affect projections over longer horizons (Methods – Model Structure, lines 128–133). We also clarify the meaning of the last term in equation (1): δ(t)I represents treated recoveries from the infectious compartment re-entering S (Methods – Model Equations, lines 125–127).

2. *Moreover, some parts of the model equations do not match the transitions shown in the transfer diagram in Fig. 2(b).*

Response: The model equations have been revised to fully match the transitions shown in Fig. 2. Missing or simplified terms (such as (1–p)σE) were restored for clarity, and vaccination flows were explicitly included in equations (1) and (2). This ensures the diagram and equations now align consistently (Methods, line 114–175).

3. *In real-world settings, vaccinated individuals are classified in a separate group, and due to imperfect vaccination, some may still become infected. The model should reflect that breakthrough infections happen directly from the vaccinated compartment, not by moving first to the susceptible compartment. In practice, we only realize the vaccine’s imperfection once vaccinated individuals become infected. Therefore, it is more appropriate for the infection to occur from the vaccinated group (V), rather than first moving them to S and then to E/I.*

Response: We have clarified in Methods that the model assumed a hypothetical sterilizing vaccine, with vaccinated individuals completely protected from infection. This simplifying assumption was intentionally adopted to highlight differences in contact structure without added complexity from waning immunity or breakthrough infections (Methods – Model Structure, line 134–139). In the Discussion, we have acknowledged this as a limitation and noted that real-world vaccines are imperfect and that breakthrough infections should be incorporated in future work [Discussion – Limitations, line 379–385).

4. *It is unclear how the two models were calibrated to produce similar dynamics despite using different values of the transmission rate β (beta)?*

Response: We have clarified in Methods that β_all was fixed at a published per-capita value, while β_net was calibrated so that baseline prevalence and incidence matched the all-to-all model. We emphasize that the parameters are not directly comparable, since one is per-capita and the other is per-contact, and that calibration is defined by aligning epidemic trajectories rather than parameter magnitudes (Methods – Parameterization and Calibration, line 189–201).

5. *The simulation technique for the network model also requires further elaboration. A clearer description is needed to help readers understand how and why the compartmental model falls short compared to the network-based implementation. The true potential of the manuscript can only be fully assessed once the identified issues are adequately addressed. I recommend that the authors revise the manuscript by resolving these concerns, providing a comprehensive and transparent description of the model and methods, and consider resubmitting the work as a new submission for further evaluation.*

Response: We have expanded the Analysis Methods subsection to describe the simulation process for the BA network model. Networks were generated using the fastnet R package, adjacency matrices handled via matrix, and outcomes simulated deterministically. We have added a plain-language explanation of how network heterogeneity produces persistence in highly connected hubs, whereas all-to-all models assume homogeneous mixing and therefore overestimate intervention impact at lower coverage levels. This elaboration clarifies how and why the network framework offers additional insights compared to the compartmental model (Methods – Analysis Methods, line 248–253; Discussion – Main Findings, line 329–345).
