# Supplementary material for: How do social network models compare to all-to-all models for forecasting tuberculosis epidemics? A mathematical modeling study
Source: PLoS One. 2026 Apr 1;21(4):e0343421. doi: 10.1371/journal.pone.0343421 (PMC13042644; doi:10.1371/journal.pone.0343421)
Supplement: Attachment — Submitted filename: Review Report PONE-D-24-57786R1.docx [file pone.0343421.s004.docx]

**Dear Editor,**

This paper is interesting having title “How do social network models compare to all-to-all models for forecasting tuberculosis epidemics? A mathematical modeling study”. The manuscript is reviewed by keeping in view the scope of the journal, and novelty.

It may be accepted for publication subject to following modifications/corrections:

1. You used a Barabási-Albert model, which is famous for creating those few ultra-connected "hubs." It made me wonder: in real-life communities, how often do social networks form exactly this way? If we used a different type of messy, clustered network (like a "small-world" one), do you think we'd still see the same strong effect, or could part of the finding be tied to this specific, very hub-heavy structure?
2. I was really struck by how different the transmission rates (β) had to be to get the models to match at the start. The network model needed a much higher per-contact risk. Does this mean that to make the epidemics look the same on the surface, we're essentially assuming that every single contact in the network is incredibly risky? And if so, does that affect how we compare them down the line?
3. Your main result is that the network model is less optimistic. Is that mostly because our interventions are like a lottery and simply miss the most connected people? Or is it that the virus has so many pathways in a network that it's just harder to block, even with a decent program? Did you get any sense of which of these forces was more dominant?
4. I totally get why you used a perfect vaccine, it keeps things clean! But it made me curious: if we imagined a more realistic, say, 60% effective vaccine, would the gap between the two models shrink, stay the same, or widen? Have you done any quick tests to see how sensitive that big finding is to the perfect-efficacy assumption?
5. Let's say I'm a health official convinced by your paper. What's my take-home? Is it that we should always add a "network reality check" to our forecasts, or is there a specific point, like when vaccine coverage is below a certain level, that this becomes absolutely critical? In other words, when should modelers like me definitely not skip this step?
6. Organize your introduction section and include latest modifications reported using nanotechnology by: <https://doi.org/10.1093/bib/bbac405>, <https://doi.org/10.1109/TCBB.2021.3059046>, <https://doi.org/10.1109/TCBB.2022.3143770>, <https://doi.org/10.1007/s00011-020-01368-4>, <https://doi.org/10.1016/j.imlet.2019.07.008>, <https://doi.org/10.1515/biol-2025-1130>
