# Supplementary material for: How do social network models compare to all-to-all models for forecasting tuberculosis epidemics? A mathematical modeling study
Source: PLoS One. 2026 Apr 1;21(4):e0343421. doi: 10.1371/journal.pone.0343421 (PMC13042644; doi:10.1371/journal.pone.0343421)
Supplement: Attachment — Submitted filename: Response to Reviewers Letter.docx [file pone.0343421.s006.docx]

**February 2, 2026**

**Re: Second Resubmission of Manuscript PONE-D-24-57786, How do social network models compare to all-to-all models for forecasting tuberculosis epidemics? A mathematical modeling study”**

Dear Dr. Mickael Essouma,

Thank you for the thoughtful and constructive feedback provided during the previous review rounds. We appreciate the opportunity to submit this second resubmission, in which we have addressed the most recent minor revision comments.

Below, we provide a point-by-point response. Reviewer comments are reproduced in italics, followed by our responses in plain text, with line numbers indicating where corresponding changes appear in the revised manuscript.

We are grateful for your continued guidance and careful consideration of our work.

Sincerely,

Masabho P. Milali, PhD

**Comments**

1. *Regarding the EPIFORGE guidelines and to avoid redundancy, details in lines 186–191 and 200–205 need to be moved to the end of the introduction where the authors state the aim of the study.*

**Response:** Thank you for this helpful suggestion. We have moved the information previously located in lines 186–191 and 200–205 to the end of the Introduction, as recommended. These changes are now reflected in lines 84–90.

2. *There is a need to revise the presentation of figures in this manuscript. For instance, the footnote of Fig 1 describes panels A and B, but I do not see those panels on Fig 1. Line 36: is it Fig 1 or Fig 2? What are Figures 4 and 6?*

**Response:** We appreciate this comment and have revised the figures for clarity and consistency.

- Figure 1 now clearly displays Panel A and Panel B, matching the caption.
- Figures 4 and 6 are now explicitly described in the text at lines 201–203 and 218–225 for Figure 4, and 292–306 for Figure 6.
- Regarding line 36, this line appears in the abstract, which does not contain figure citations. Figure 1 is described in lines 99–110, and Figure 2 in lines 148–152.

3. *Fig. 3, 4, 5, and 6 should be clearly specified each (including any intrinsic panels) with its footnote and title together as you did for Fig 1 or Fig 2.*

**Response:** As suggested, we have revised and standardized the titles and footnotes for Figures 3, 4, 5, and 6, ensuring that each figure clearly specifies any intrinsic panels and follows the same structure as Figures 1 and 2. These updates appear in lines 165–171, 218–225, 283–290, and 297–306.

4. *Could you specify the published literature you are mentioning in line 293?*

**Response:** Thank you for this observation. The relevant published literature has now been explicitly cited in line 86, following your earlier suggestion to relocate the content from lines 186–191 and 200–205 to the end of the Introduction where we state the study aims.

5. *Did the End TB by 2035 strategy inform the target figures for treatment and vaccination used in this study or were they set arbitrarily? This should be addressed in the discussion as well.*

**Response:** Thank you for highlighting this important point. The intervention levels used in our analysis (vaccination coverage of 30–70% and treatment improvements of 20–50%) were not derived from the WHO End TB Strategy 2035 operational targets, which focus on long-term reductions in incidence, mortality, and catastrophic costs. Instead, these values were selected as exploratory ranges commonly used in transmission modeling studies to examine the influence of incremental improvements in prevention and treatment. Their purpose was to isolate how contact structure modifies intervention impact while maintaining comparability across scenarios. We have added a clear explanation of this rationale in the Discussion (lines 361–370).
