# Supplementary material for: How do social network models compare to all-to-all models for forecasting tuberculosis epidemics? A mathematical modeling study
Source: PLoS One. 2026 Apr 1;21(4):e0343421. doi: 10.1371/journal.pone.0343421 (PMC13042644; doi:10.1371/journal.pone.0343421)
Supplement: Attachment — Submitted filename: Do social networks attenuate the population Referee report.pdf [file pone.0343421.s002.pdf]

Do social networks attenuate the population-level impact of tuberculosis interventions? A mathematical modeling study

Referee report

This study investigates whether social networks diminish the projected impact of tuberculosis (TB) interventions using mathematical modeling. Researchers developed two TB transmission models with identical assumptions—one assuming "all-to-all" mixing (where every individual can infect any other) and the other using a Barabási-Albert scale-free social network (where infection occurs only among social contacts).

Interventions including vaccination and treatment were simulated across varying coverage and efficacy levels. The social network model consistently projected lower reductions in TB incidence, especially under low-coverage or low-efficacy scenarios. For instance, a vaccine with 30% coverage and full efficacy reduced cases by 72% in the network model versus 94% in the all-to-all model. Similarly, modest treatment efficacy improvements yielded significantly smaller impacts in the network-based approach. These differences persisted across varying population sizes and network densities. The study concludes that traditional all-to-all models may overestimate the benefits of sub-optimal interventions and thus influence unrealistic target-setting in TB control strategies.

Although high-efficacy and high-coverage interventions showed consistent impact across both models, the inclusion of network structures is crucial in forecasting TB trends more accurately. Limitations include generalized assumptions, single network type, and simplifications in vaccine modeling. The authors call for expanded modeling efforts incorporating real-world social network complexities.

I find that this study is of real interest and has the merit to be considered for publication. Its primary strength lies in its rigorous head-to-head comparison of two modeling paradigms under consistent disease dynamics and intervention parameters. The use of a Barabási-Albert scale-free network brings realism to contact patterns and highlights the role of heterogeneity in TB transmission. It challenges common assumptions in TB modeling and stresses caution in policy planning and its findings are robust across multiple scenarios and offer valuable insights into the implications of model structure on forecasting and public health decision-making.

However, I also note that there are several issues that may need to be addressed for further improvement. I recommend that the authors revise their manuscript while addressing each of the following points:

- The model is not tailored to specific geographic or demographic settings. Can the authors relate their work to real world epidemiological data?
- The authors should include in the discussions section a few lines about the possibility of exploring additional network topologies (e.g., small-world or community-based) to explain how structural differences influence intervention outcomes.

- Instantaneous vaccination is an issue of concern in the model. Real life scenarios would have gradual roll-out of vaccination. I suggest to address this issue as well in the discussions.
- The issue of vaccine availability as well as timing of introduction is discussed and modelled in the literature. One such study that the authors can refer to on their introduction or in their discussion while addressing the previous points is the following: https://doi.org/10.1371/journal.pone.0267840
- Assumption of 100% vaccine efficacy is very ideal and usually not true. I suggest that in the model results,the authors consider a bracket of efficacies, for example 80-85-90-95% scenarios, with corresponding plots and comparisons. This could be done 1 figure, then move to a particular benchmark in the rest of the results, maybe 90% per se.
- Social determinants affecting treatment uptake and exposure risk were not considered. Explain how such factors could improve projections and guide equity-oriented interventions.

I recommend a major revision to specifically address these points, and, if treated well, the paper would be suitable for consideration afterwards.
